# Tumor microenvironment disparity in multiple primary lung cancers: Impact of non-intrinsic factors, histological subtypes, and genetic aberrations

**DOI:** 10.1016/j.tranon.2021.101102

**Published:** 2021-04-27

**Authors:** Motohiro Izumi, Kenji Sawa, Jun Oyanagi, Ikue Noura, Mitsuru Fukui, Koichi Ogawa, Yoshiya Matsumoto, Yoko Tani, Tomohiro Suzumura, Tetsuya Watanabe, Hiroyasu Kaneda, Shigeki Mitsuoka, Kazuhisa Asai, Noritoshi Nishiyama, Masahiko Ohsawa, Nobuyuki Yamamoto, Yasuhiro Koh, Tomoya Kawaguchi

**Affiliations:** a Department of Respiratory Medicine, Graduate School of Medicine, Osaka City University, Osaka, Japan; b Internal Medicine III, Wakayama Medical University, 811-1 Kimiidera, Wakayama-shi, Wakayama 641-8509, Japan; c Department of Pathology, Graduate School of Medicine, Osaka City University, Osaka, Japan; d Laboratory of Statistics, Graduate School of Medicine, Osaka City University, Osaka, Japan; e Department of Clinical Oncology, Graduate School of Medicine, Osaka City University, Osaka, Japan; f Department of Thoracic Surgery, Graduate School of Medicine, Osaka City University, Osaka, Japan

**Keywords:** Tumor microenvironment, Multiple primary lung cancer, Non-intrinsic factor, Histology, Genetic aberration, PD-1, programmed cell death-1, PD-L1, programmed death-ligand 1, NSCLC, non-small cell lung cancer, ICI, immune checkpoint inhibitor, TME, tumor microenvironment, TIL, tumor-infiltrating lymphocyte, NGS, next-generation sequencing, TMB, tumor mutation burden, MPLC, multiple primary lung cancer, AIS, adenocarcinoma in situ, MIA, minimally invasive adenocarcinoma, TC, tumor cell, IC, immune cell

## Abstract

**Introduction:**

Multiple primary lung cancers (MPLCs) occur in common carcinogenetic risks such as lifestyle, biological aging, immune responses, hormones, and metabolism. Although MPLCs harbor various genetic profiles within the same individuals, differences in the tumor microenvironment (TME) are unclear. We investigated the impact of genetic aberrations, non-intrinsic factors, and pathological subtypes on tumor immunity.

**Materials and Methods:**

In total, 73 surgically resected specimens from 32 patients with MPLC were analyzed. PD-L1 expression in tumor cells (TCs) and immune cells (ICs), CD3-positive tumor-infiltrating lymphocytes (TILs), CD8/CD3 ratios, and FOXP3-positive TILs that compose TMEs were evaluated by immunohistochemistry and classified on a score of 0–2. 38 tumors were sequenced for somatic mutations in 409 cancer-associated genes.

**Results:**

Females and never or light smokers had a higher incidence of PD-L1-negative tumors and a higher concordance rate. PD-L1 positivity in TCs and ICs was significantly less frequent in *EGFR*-mutated than in wild-type tumors. Differences in the score of TMEs were observed between the *KRAS*-mutated-only tumor and the *KRAS* and *TP53*-co-mutated tumors, and between the *KRAS*-mutated-only tumor and the *KRAS* and *STK11*-co-mutated tumors. Significantly more FOXP3-high TILs were observed in invasive pathological subtypes than in non-invasive ones.

**Conclusion:**

Comparing TMEs among MPLCs revealed that non-smokers or light smokers and females were unlikely to express PD-L1 regardless of tumor site and confirmed that the *EGFR* mutations and co-occurring *KRAS* and *STK11* or *TP53* mutations were associated with TME. Pathological subtypes may impact the efficacy of immune therapy due to their potential correlations with regulatory T cells.

## Highlights

• Tumor microenvironment (TME) was compared among multiple primary lung cancers (MPLCs).
• Sex and smoking status concomitantly impacted PD-L1 expression in paired tumors.
• *EGFR* mutations were independently associated with PD-L1 expression.
• *KRAS* mutations altered the TMEs according to the types of co-mutations.
• The number of FOXP3-positive t cells reflected histological subtypes.

## Introduction

Lung cancer is the leading cause of cancer-related deaths worldwide [1]. The development of immunotherapy targeting programmed cell death-1 (PD-1) and programmed death-ligand 1 (PD-L1) has revolutionized the treatment of advanced non-small cell lung cancer (NSCLC) [2], [3], [4], [5], [6], [7], [8], [9], [10]. Furthermore, immunotherapy is expected to be useful in adjuvant or neoadjuvant settings, and a number of trials are currently evaluating whether immune checkpoint inhibitors (ICIs) can be incorporated into the care of patients with early stage NSCLC [11,12]. Although previous studies have indicated that the cell-surface expression of PD-L1 protein is an effective biomarker for predicting the response to ICIs [13], it does not consistently represent a reproducible predictive biomarker of the clinical response [14], [15], [16]. Other components of the tumor microenvironment (TME), such as cytotoxic CD8-positive tumor-infiltrating lymphocytes (TILs) and regulatory T cells, critically contribute to tumor biology and the therapeutic response to ICIs [17,18]. In addition, epigenetic analysis based on DNA methylation can be a diagnostic tool for early detection of cancers [19], and PD-L1 promoter methylation induced by ICIs might be related with resistance to anti-PD-1 therapy [20]. They suggest that predictive biomarkers aimed at personalized therapy can be identified by a coordinated multiparametric definition of the immune contexture [21,22].

Cancer risk factors can be divided into intrinsic factors and non-intrinsic factors. Intrinsic factors are defined as unmodifiable intrinsic DNA replication errors that can occur with a certain probability during cell division. Non-intrinsic factors are risks other than intrinsic replication error and include modifiable exogenous/external factors, such as lifestyle factors, that are exogenous to the host, and endogenous factors that are partially modifiable and related to the characteristics of an individual and influence key aspects of cell growth control and genome integrity. The majority of cancer risk factors are non-intrinsic [23,24]. These non-intrinsic factors have been indicated as factors affecting TME. It has been demonstrated that PD-L1 positivity in NSCLC samples is associated with current smoking status [25]. Generally, tumors in continuous smokers have more somatic mutations compared with tumors in those who have never smoked [26]. Targeted next-generation sequencing (NGS) accurately estimates tumor mutation burden (TMB), and elevated TMB further improves the likelihood of benefitting ICIs. There have been conflicting results of no significant association between TMB and age, sex, smoking history, or prognosis in early stage NSCLC [27]. Both TMB and PD-L1 expression have similar predictive capacities for ICIs, but no correlation between them has been demonstrated [28]. Therefore, non-intrinsic factors, including smoking history and its influence on the effect of ICIs, are somewhat divergent.

It has also been indicated that genetic aberrations and pathologic subtypes play an important role in TMEs. In NSCLC samples, a significantly higher proportion of PD-L1-positive expression occurs in *EGFR* wild-type tumors compared to *EGFR*-mutated counterparts [29]. Furthermore, decreased TMB, lack of TILs, and reduction in the proportion of PD-L1-positive/CD8-positive TILs have been observed in *EGFR*-mutated tumors [30]. However, the existence of several subtypes of *EGFR*‐mutated NSCLC according to the TME, based on tumor expression of PD‐L1 and CD8-positive TILs, and the efficacy of EGFR‐TKIs differs according to the TME [31]. *STK11* inactivation is a negative predictive biomarker of the response to ICIs [32]. In terms of histology, PD-L1 expression has been associated with solid subtype histology, epithelial-mesenchymal transition, and poor prognosis in lung adenocarcinomas, but not in squamous cell lung cancers [33]. Plasma and B cells with interfollicular distribution have been almost exclusively observed in invasive histologic subtypes, while an increased number of mast cells have been observed in noninvasive histologic subtypes [34].

The variability of PD-L1 expression among and within NSCLC patients is intrinsic to several biological and genetic events implicated in tumor immunogenic properties [26]. In this regard, neoantigen generation and timing of the natural immune history largely vary among patients, and markedly impact PD-L1 expression [35]. In addition, due to the intricate link between various carcinogenic risk factors and TMEs constituting various immune-related cells, studies comparing patients with different backgrounds have limitations. Therefore, in the present study, we focused on multiple primary lung cancers (MPLCs) that occur in patients exposed to identical risk factors and systemic reactions, including the immune response. MPLC is diagnosed based on pathological findings such as histologic subtypes, and radiological findings such as tumor shape, location, and lymphadenopathy in clinical. We hypothesized that the TME would make the same changes in multiple lesions within the same individuals if non-intrinsic factor-related systemic reactions were strongly associated with the TME. We speculated that genetic aberrations and pathological subtypes would impact the disparity of TMEs among MPLCs, even within the same individuals. In this study, we compared TMEs among MPLCs to elucidate how various factors impact tumor immunity.

## Materials and methods

### Study population

In the present retrospective study, we identified 78 tumors from 34 patients who underwent surgical resection for MPLCs at Osaka City University between May 2007 and March 2019. In addition to the clinicopathological features, patient age, sex, smoking history, body mass index, location of tumors, and surgical procedures were obtained. All available information was carefully reviewed, and intrapulmonary metastasis was excluded by a multidisciplinary tumor board that consisted of radiologists, thoracic surgeons, and medical oncologists. Written informed consent forms were obtained from all patients, and the study was approved by the institutional review board at Osaka City University Hospital and Wakayama Medical University Hospital.

### Diagnostic approach

We diagnosed MPLC based on the methods described in previous studies [36], [37], [38]. MPLC was diagnosed if (1) paired nodules were located in different segments, lobes, or lungs; (2) either of the paired nodules was adenocarcinoma in situ (AIS) or minimally invasive adenocarcinoma (MIA); (3) the histopathologic patterns, including subtypes, were different between paired nodules; (4) nodal or systemic metastases were absent; or (5) CT imaging showed the presence of an air-bronchogram or speculation in paired nodules. In addition, a cancer-free interval of at least two years was diagnosed as metachronous MPLC, whereas synchronous MPLC consisted of a cancer-free interval of less than two years.

### Immunohistochemistry (IHC)

IHC was performed using successive sections from formalin-fixed paraffin-embedded specimens at N Lab (Nagasaki, Japan). The tumor area was defined as the one containing at least 100 viable tumor cells (TCs) and their associated intratumoral stroma and contiguous peritumoral stroma. AIS, MIA, and lepidic adenocarcinomas were defined as noninvasive pathological subtypes and other histologies, including papillary, acinar, solid, adenosquamous, and squamous cell carcinomas, as invasive pathological subtypes. PD-L1 expression was assessed or scored independently by two pathologists at the N Lab, and the number of CD3-, CD8-, and FOXP3-positive TILs was assessed by two pathologists at Osaka City University Hospital. If the independent assessments were not in agreement, the slides were reviewed together by both investigators until they reached a consensus. Consensus judgments were adopted as the final results.

PD-L1 expression was evaluated with a rabbit monoclonal antibody (mAb) (dilution 1:1200, clone 28–8, Abcam Cat# ab205921, RRID: AB_2,687,878). PD-L1 expression in TCs was assessed as the proportion of TCs showing membrane staining of any intensity, and expression in immune cells (ICs) was assessed as the proportion of tumor area occupied by PD-L1-positive ICs of any intensity. We classified PD-L1 expression in TCs as score 0 (< 1%), score 1 (1‐49%), or score 2 (≥ 50%), and PD-L1 expression in ICs as score 0 (< 1%), score 1 (1‐9%), or score 2 (≥ 10%) (Supplementary Table 1, Supplementary Fig. 1).

The presence of CD3-, CD8-, and FOXP3-positive TILs was assessed by IHC staining using mouse mAb (CD3, dilution 1:50, Agilent Cat# M7254, RRID: AB_2,631,163; CD8, dilution 1:400, Agilent Cat# M7103, RRID: AB_2,075,537; FOXP3, dilution 1:50, Abcam Cat# ab20034, RRID: AB_445,284) in TCs and in tumor stroma, where TC invasion was observed. The number of CD3-, CD8-, and FOXP3-positive TILs was counted at each of the four random areas over a range of 0.23. 0.31 mm on the whole tumor specimen separately for invasive and non-invasive pathological subtypes using Imaging Software NIS-Elements D 3.2 (Nikon). The number of cells counted was adjusted to the number of cells per 0.22 mm^2^. The cut-off values of the CD3-positive TILs, CD8/CD3 ratios, and FOXP3-positive TILs were determined as reported in a previous study [34] (Supplementary Table 1, Supplementary Fig. 2), and we defined score 2 as “high” and scores 0 or 1 as “low”.

### Library preparation and sequencing

NGS for the detection of actionable somatic mutations was carried out as previously described [39]. Briefly, sequencing was carried out on an Ion GeneStudio S5 (Thermo Fisher Scientific) for somatic mutations in 409 cancer-associated genes (Ion AmpliSeq Comprehensive Cancer Panel) (Supplementary Table 2). The TMB was calculated as the number of non-synonymous somatic, coding, base substitution, and indel mutations per mega-base (Mb).

The data for samples that were not eligible for NGS but were tested for *EGFR* mutations in a clinical setting using the cobas® EGFR Mutation Test (Roche Molecular Systems) were included.

### Statistical analysis

Comparisons of categorical data were analyzed using chi-squared tests, except when a small sample size (< 5) required the use of Fisher's exact test. In cases with more than three lesions, it was considered discordant if none of the lesions showed the same TME when the concordance rate was analyzed. All *P* values were based on a two-tailed test, and results with *P* value of less than 0.05 were considered statistically significant.

## Results

### Patient characteristics

**Fig. 1:**
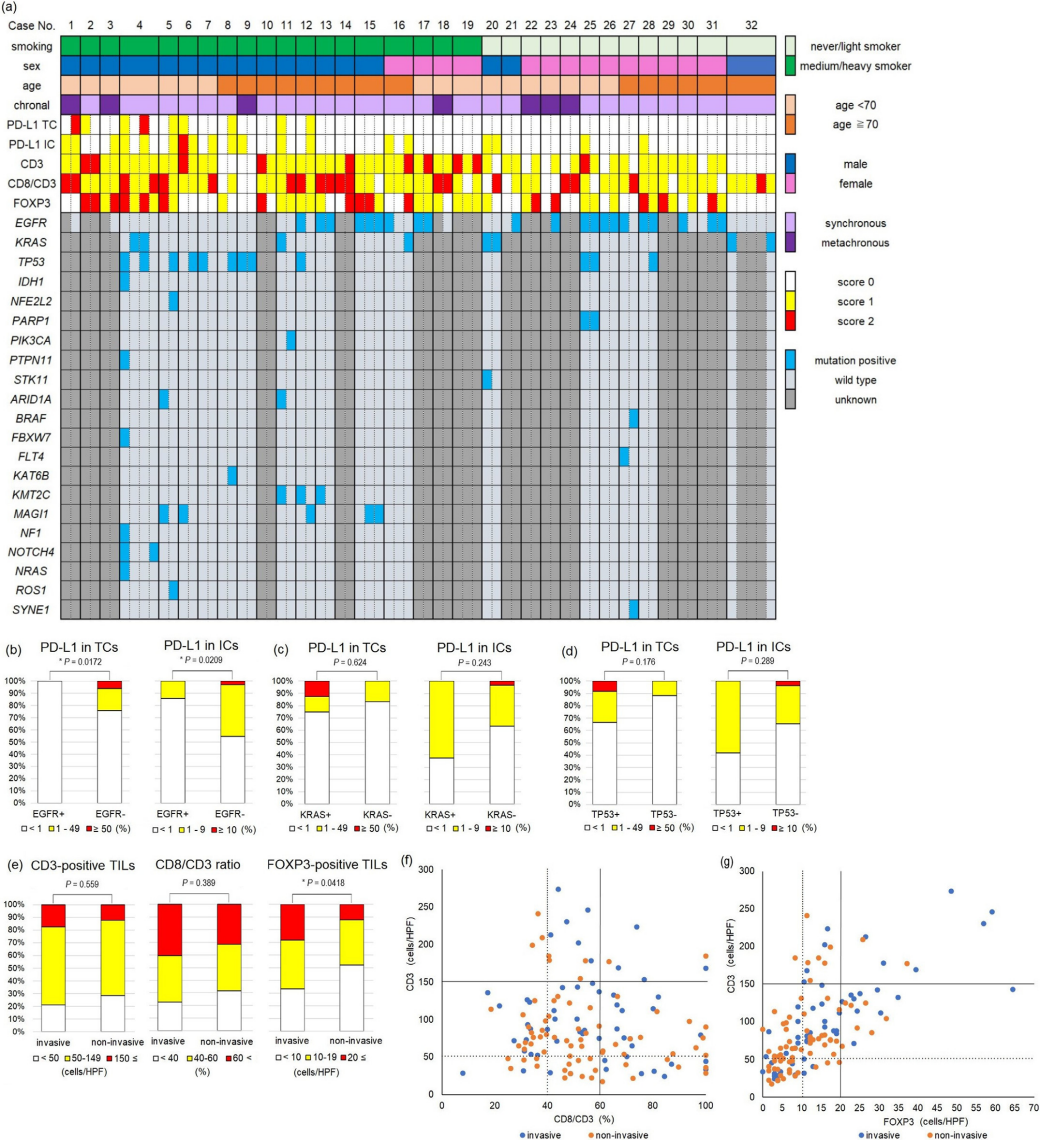
Relationship of tumor microenvironment with genetic aberrations and histological subtypes. (a) Tumor microenvironment and mutation profiling in multiple primary lung cancers. Characteristics and scores of PD-L1 expression in TCs, PD-L1 expression in ICs, CD3-positive TILs, CD8/CD3 ratio, FOXP3-positive TILs, and mutation profiling were visualized. Correlation between PD-L1 expression and (b) *EGFR*, (c) *KRAS*, and (d) *TP53* mutations. (e) Correlation between pathological subtypes and CD3-positive TILs, CD8/CD3 ratio, and FOXP3-positive TILs. (f) A scatter plot of CD3-positive TILs and the CD8/CD3 ratio according to the pathological subtypes showed that plots were not biased regardless of histology. (g) A scatter plot of CD3- and FOXP3-positive TILs according to the histological subtypes showed that invasive pathological subtypes were biased toward high FOXP3. The dotted line indicates the lower cut-off value, and the straight line indicates the higher cut-off value. TC; tumor cell, IC; immune cell, TIL; tumor infiltrating lymphocyte, TIL low scores; 0 or 1, TIL high score; 2. An asterisk (*) indicates *P* < 0.05.

We obtained 78 surgical specimens from 34 patients who were diagnosed with MPLC and underwent surgery at Osaka City University Hospital between October 2007 and March 2019. Among them, 73 specimens from 32 patients were eligible for immunohistochemical analysis (Supplementary Fig. 3). Additionally, deep sequencing was successfully performed in 38 tumors from 17 patients. We defined light smokers as Brinkman Index (BI) <200, medium smokers as BI 200 to 600, and heavy smokers as BI >600 in accordance with a previous report [40]. The background information and clinical data of 32 patients involved in this study are shown in Table 1. Patient ages ranged from 50 to 83 years (mean age: 68 years). The patients consisted of 18 males and 14 females, and 19 ever-smokers and 13 non-smokers. The characteristics, TME, and mutation profiling of each lesion are summarized in Fig. 1(a). Tumor location, pathological stage, maximum diameter of the tumors, and histology are shown in Supplementary Table 3.

**Table 1:** Patient characteristics.

BMI, body mass index; MPLC, multiple primary lung cancer.

### Positivity and concordance rate in the TME

Histology subtype classification, TME score, and representative genetic mutations are shown in Table 2. PD-L1-positivity in TCs and ICs was observed in nine (12.3%) and 19 (26.0%) tumors, respectively. Among the 32 patients with MPLC, concordance was observed with PD-L1 expression in the TCs of 24 patients (75.0%), ICs in 20 patients (62.5%), CD3-positive TILs in 18 patients (56.2%), CD8/CD3 ratios of 17 patients (53.1%), and FOXP3-positive TILs in 14 patients (43.7%) (Table 3). There was a significant difference in the concordance rate of the CD8/CD3 ratio between patients with synchronous and metachronous MPLCs, and the other TME had no correlation with chronal difference (PD-L1 expression in TCs, *P* = 0.646; PD-L1 expression in ICs, *P* = 1.000; CD3-positive TILs, *P* = 1.000; CD8/CD3 ratio, *P* = 0.00769; FOXP3-positive TILs, *P* = 1.000) (Table 3).

**Table 2:** Classification of histological subtype, score of tumor microenvironment, and genetic aberration in each tumor. AIS, adenocarcinoma in situ; MIA, minimally invasive adenocarcinoma; Sq, squamous cell lung carcinoma; TIL, tumor infiltrating lymphocyte; WT, wild-type.

**Table 3:** Concordance rate in each characteristic. (a) Concordance rate of PD-L1 expression in TCs and ICs. (b) Concordance rate of CD3-positive TILs, CD8/CD3 ratio, and FOXP3-positive TILs. Values are n (%). ICs, immune cells; TCs, tumor cells; TILs, tumor infiltrating lymphocytes.

### Relationship between non-intrinsic factors and the TME

We analyzed 73 tumors to elucidate the relationship between non-intrinsic factors, such as age, sex, smoking status, and TME. PD-L1 positivity in TCs was significantly more frequent in males and medium or heavy smokers (*P* = 0.00821 and 0.00855, respectively), and PD-L1 positivity in ICs was significantly more frequent in males (*P* = 0.0335) (Table 4). CD3-positive TIL scores were significantly higher in medium or heavy smokers than in never or light smokers (*P* = 0.00210) (Table 4). There was no significant difference between the other non-intrinsic factors and TIL scores.

**Table 4:** Correlation between characteristics and tumor microenvironment. (a) Positivity of PD-L1 expression in TCs and ICs in each characteristic. (b) TILs high or low for each characteristic. Values are presented as n (%). IC, immune cells; TC, tumor cells; TILs, tumor-infiltrating lymphocytes; PD-L1 negative, PD-L1 expression less than 1%; PD-L1 positive, PD-L1 expression more than 1%; low TILs, score 0 or 1; high TILs, score 2.

Next, we analyzed 32 cases consisting of paired tumors to evaluate whether non-intrinsic factors caused the TME to change similarly in multiple tumors within the same individual. There were no cases with concomitant PD-L1 positive TCs, and only two cases with concomitant PD-L1 positive ICs within the same individuals. The concordance rate of PD-L1 expression in TCs was significantly lower in males and medium or heavy smokers (*P* = 0.00445 and 0.0104, respectively), and the rate of PD-L1 expression in ICs was significantly lower in males (*P* = 0.00742) (Table 3). There were few cases with a concomitant score of 0 or 2 for CD3-positive TILs, CD8/CD3 ratio, and FOXP3-positive TILs in multiple lesions within the same individuals (Table 3). Any non-intrinsic factors, including age, sex, and smoking status, had little impact on these TILs.

### Correlation of genetic aberrations in the TME

We analyzed the samples as single tumors to evaluate whether *EGFR, KRAS*, and *TP53* mutations detected in multiple cases using NGS or the cobas® EGFR Mutation Test were associated with the TME (Fig. 1(b)–(d), Supplementary Table 4, Supplementary Fig. 4). *EGFR* mutations were tested in 54 lesions*, and KRAS* and *TP53* mutations were tested in 38 lesions. PD-L1 positivity in both TCs and ICs was significantly lower in *EGFR*-mutated tumors than in wild-type tumors (*P* = 0.0172 and 0.0209, respectively). *EGFR* mutations were not correlated with CD3-positive TILs, CD8/CD3 ratio, and FOXP3-positive TILs. *KRAS* and *TP53* mutations were not correlated with TME.

### Correlation of pathological subtypes in the TME

The results showing the evaluation of the TME by classifying pathological findings into invasive and non-invasive lesions are shown in Fig. 1(e)–(g). Invasive subtypes were detected in 57 lesions, while noninvasive subtypes were detected in all 73 lesions. There was no significant difference among pathological findings regarding CD3-positive TILs and the CD8/CD3 ratio (*P* = 0.559 and 0.389, respectively). FOXP3-high TILs were observed more frequently in invasive lesions than in non-invasive lesions (*P* = 0.0418).

### TMB

In this study, TMB was measured using NGS on 409 cancer-associated genes. The overall median TMB was 0.59 mutations/Mb, ranging from 0 to 4.1 mutations/Mb (Supplementary Fig. 5). Although there were variations, TMB in all lesions showed less than 5 mutations/Mb. There were no characteristic TMB changes due to non-intrinsic factors or pathological subtypes.

### Case presentations

Three interesting cases are presented below in detail.

**Fig. 2:**
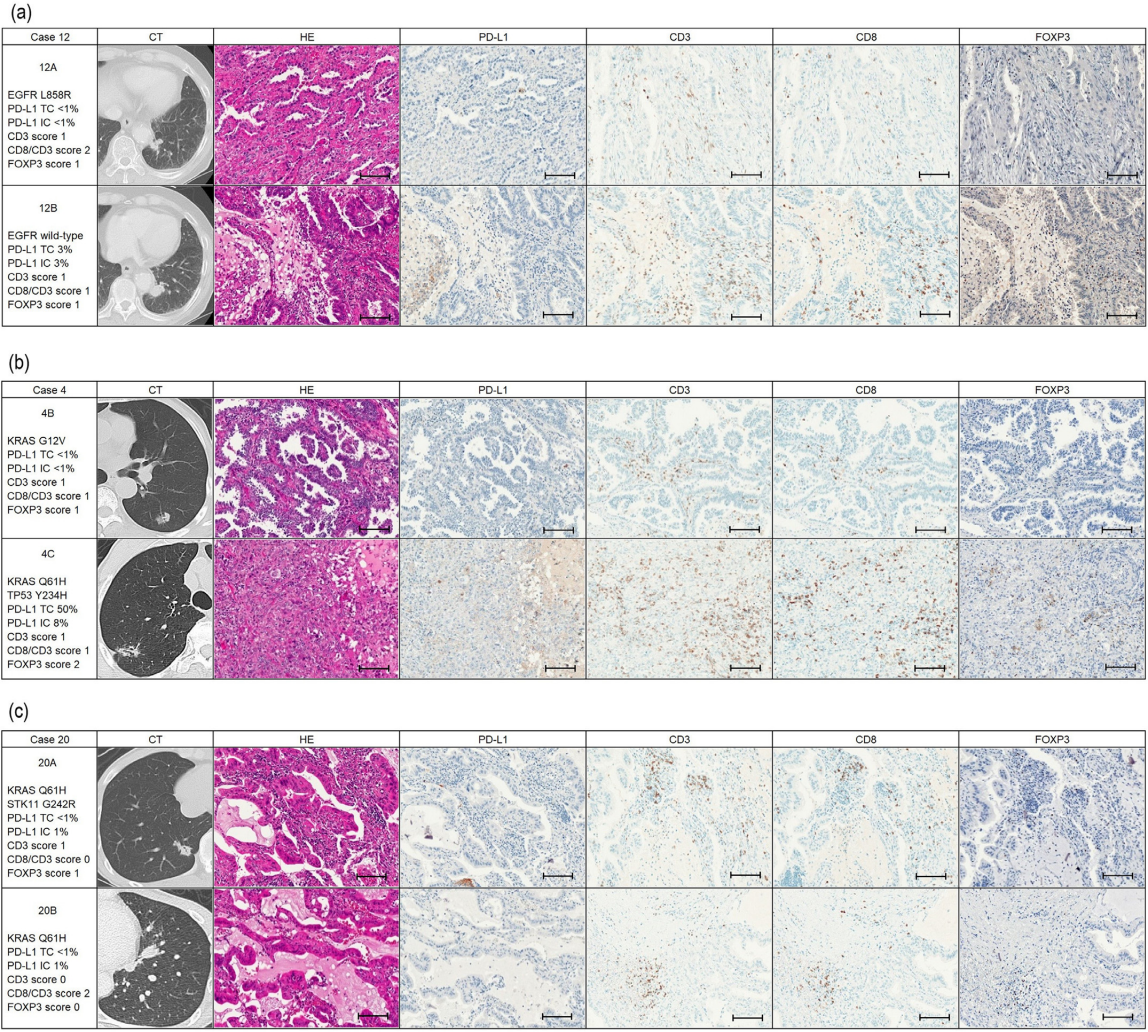
Summary of cases according to radiological and pathological examinations. Differences of the tumor microenvironment according to mutation profiling shown in (a) Case 12 which had *EGFR*-mutated and wild type tumors, (b) Case 4 which had *KRAS*-only-mutated and *KRAS* and *TP53*-comutated tumors, (c) Case 20 which had *KRAS*-only-mutated and *KRAS* and *STK11*-commutated tumors. Scale bar = 100 μm.

Case 12: An 83-year-old male heavy smoker arrived at our department after computed tomography (CT) detected two masses adjacent to each other in the left S10. There was no significant lymphadenopathy or metastases, so a left lower lobectomy was performed. Pathologically, both tumors were classified as predominantly papillary adenocarcinomas. However, one tumor (12A) had *an EGFR* L858R mutation and lepidic components, whereas the other tumor (12B) had wild-type *EGFR* and relatively large amounts of acinar components without a lepidic component. Differences in the TMEs were observed between the 12A tumor (PD-L1 expression in TC < 1%, PD-L1 expression in IC < 1%, CD3 score 1, CD8/CD3 score 2, and FOXP3 score 1) and the 12B tumor (PD-L1 expression in TC 3%, PD-L1 expression in IC 3%, CD3 score 1, CD8/CD3 score 1, and FOXP3 score 1) (Fig. 2(a)). The TME, especially PD-L1 expression, was changed by *EGFR*-mutated or wild-type tumors even within the same segments.

Case 4: A 67-year-old male medium smoking patient arrived at our department after four masses were detected in the left S3, left S6, right S2, and right S6 lesions on a chest CT performed as part of a detailed examination for a cough. A CT image showed a ground-glass opacity-predominant part-solid nodule, air-bronchogram, and spiculation in all lesions. The left S6 tumor was classified as a predominantly papillary adenocarcinoma, whereas the right S2 tumor was classified as a solid adenocarcinoma. Mutation profiling and immune status differed between them. In particular, there was a difference in PD-L1 expression and the score of FOXP3-positive TILs between the S6 lesion, which was the *KRAS*-mutated-only tumor, and the right S2 lesion, which was the *KRAS* and *TP53*-co-mutated tumors (PD-L1 expression in TCs, less than 1% and 50%; PD-L1 expression in ICs, less than 1% and 8%; FOXP3-positive TILs, scores 1 and 2, respectively) (Fig. 2(b)). Differences in PD-L1 expression were observed in the presence of co-mutations despite both tumors occurring in the same smoking patient and harboring the *KRAS* mutation.

Case 20: A 50-year-old male who had never smoked had nodules in the right and left S10 on a computed tomography (CT) scan to follow-up gallbladder adenomyosis. These two lesions were located in different lungs, and no significant lymphadenopathy was common. Therefore, he underwent a basal segmentectomy for both. Pathologically, both tumors were classified as predominantly papillary adenocarcinoma, but the patient had no recurrence for more than five years; thus, this case is consistent with MPLC. The right S10 tumor harbored *KRAS* and *STK11*-co-mutations, CD3-positive TILs of score 1, CD8/CD3 ratio of score 0, and FOXP3-positive TILs of score 1, whereas the left S10 tumor harbored only the *KRAS* mutation, CD3-positive TILs of score 0, CD8/CD3 ratio of score 2, and FOXP3-positive TILs with a score of 0 (Fig. 2(c)). *KRAS* mutations showed different TMEs according to the presence or absence of *STK11*.

## Discussion

This study evaluated the TMEs of MPLCs and demonstrated that sex and smoking status impacted PD-L1 expression. Additionally, *EGFR* mutations were independently associated with PD-L1 expression, whereas *KRAS* mutations appeared to alter the TME according to the type of co-mutation. The differences in the number of FOXP3-positive TILs reflected the histological subtypes.

PD-L1 expression is a biomarker for the efficacy of ICIs, but a previous study proposed the stratification of the TME into four different types based on the presence or absence of TIL and PD‐L1 expression to predict the response to ICIs [41]. Numerous IC types and components are related to cancer immunity. In addition, the TME is influenced by various factors, including non-intrinsic factors, genetic aberrations, and histologic subtypes.

We considered focusing on MPLCs and comparing the TME among tumors that occurred in the same background to enumerate which risk factors impacted the TME. It is crucial to differentiate MPLC from intrapulmonary metastasis. The criteria based on tumor locations and pathological findings used to define MPLCs were initially published in 1975 [36]. Additionally, a novel algorithm to differentiate between MPLCs and intrapulmonary metastasis using clinical and imaging variables has been reported [38]. The algorithm showed combinations of lesion types on CT, and the presence of air-bronchogram and spiculation were the most accurate determinants for differentiation between MPLC and intrapulmonary metastasis. We thoroughly reviewed all available information, including the locations of the tumors, air-bronchogram, spiculation, lymph nodes or distant metastasis, pathologic subtypes, and clinical course to exclude intrapulmonary metastasis.

The differences in TMEs among MPLCs are not well known. The concordance rate of PD-L1 expression in TCs was 75.0%, which is consistent with the results of a previous study [42], and there was no significant difference between synchronous and metachronous MPLCs (72.0% and 85.7%, respectively). Tobacco smoking patients with NSCLC generally have a higher PD-L1 expression and exhibit a better overall response rate to ICIs than those who have never smoked, whereas the relationship between PD-L1 expression and the other non-intrinsic factors, including age and sex, was not demonstrated. Our study, using MPLC samples, also showed that medium or heavy smokers had more PD-L1-positive tumors. However, there were no cases of PD-L1-positivity in paired lesions, even within the same smoker individuals. Tar and nicotine have immunosuppressive effects on the innate immune response, and tobacco products containing high concentrations of tar and nicotine cause maximum immunological changes [43]. Additionally, it has been reported that cigarette smoke and the carcinogen benzo(a)pyrene induce PD-L1 expression in lung epithelial cells both in vitro and in vivo, which is mediated by the aryl hydrocarbon receptor [44]. Our results indicate that the effects of tobacco on immunity are not uniform*.* In addition, both the frequency of PD-L1-negative tumors and concordance rate of PD-L1 expression in TCs and ICs were higher in females than in males. *EGFR* negative tumors and medium or heavy smokers were also present in the females. Therefore, our study demonstrated the relationship between sex and PD-L1 expression.

The concordance rate of the CD8/CD3 ratio was different between synchronous and metachronous MPLCs. This indicates that CD8-positive TILs may change over time. There was no correlation between TILs and age or sex. The difference in CD3 score was shown by smoking status, but the concordance rate of the CD3 score among MPLCs was low, so smoking status had a low impact on CD3-positive TILs. A previous study showed that FOXP3-positive TILs were age-related, indicating that the phenomenon of immunosenescence and the changes in immune responses were related to age [45]. However, it is possible that the differences in cut-off values and age graphics reflect the discrepancies in the results.

In contrast to preclinical studies [46], clinical evidence suggests that *EGFR*-mutated lung cancers rarely benefit from treatment with ICIs [47]. All tumors with *EGFR* mutations in this study were PD-L1 negative, and disparity of PD-L1 expression was observed among multiple lesions by the presence or absence of *EGFR* mutations, such as Case 12*,* which involved two tumors with similar pathological subtypes within the same segment. We further revealed that the *EGFR* mutation strongly affected PD-L1 expression using MPLC samples. Subsets of *KRAS*-mutated lung adenocarcinomas by co-occurring genetic events in *STK11/LKB1* or *TP53* differ biologically and therapeutically. In agreement with lower PD-L1 expression in TCs, co-occurring *KRAS* and *STK11/LKB1* subgroups exhibited lower densities of CD3- and CD8-positive but not FOXP3-positive TILs [48]. In contrast, *KRAS* and *TP53*-co-mutated tumors demonstrated higher levels of somatic mutations, inflammatory markers, immune checkpoint effector molecules, and improved relapse-free survival. Several targetable mediators of cell-intrinsic coinhibitory signals were expressed at higher levels in the co-occurring *KRAS* and *TP53* cluster, including PD-L1, PD-1, and CTLA-4, and there was a trend toward denser infiltration with CD3- and CD8-positive populations of lymphocytes [32]. In this study, one case involved one tumor harboring only a *KRAS* mutation, and the other co-occurred as *KRAS* and *STK11*. CD3-positive TILs, CD8/CD3 ratio, and FOXP3-positive TILs differed between the two tumors. Co-occurrence of *KRAS* and *STK11*-co-mutated tumors showed that PD-L1 expression in TCs was negative, and CD8-positive TILs were low, suggesting immunotherapy-resistant TME, and these results were consistent with those of previous studies [32]. Another case involved the *KRAS-*only mutated tumor and *KRAS* and *TP53* co-mutated tumors. The tumor with only the *KRAS* mutation showed negative PD-L1 expression in both TCs and ICs, whereas the *KRAS* and *TP53*-co-mutated tumor showed that PD-L1 in TCs was highly expressed, and the FOXP3-positive TIL score was high. The case showed different TMEs despite these tumors occurring within the same individuals. In addition to the effect of smoking resulting in non-uniform PD-L1 expression and inflammatory responses within the same individuals, co-occurring *KRAS* and *TP53* mutations induced by inflammatory responses in one of paired tumors also might confer non-uniform tumor immunity such as PD-L1 expression. We could perform international validation of the correlation between co-occurring genetic events and TMEs using samples with MPLC although further investigation in a larger cohort is desirable. The present study comparing mutation profiles with TMEs among MPLCs clarified that *EGFR* mutations affect TMEs by a single mutation, while some co-occurring mutations, such as *KRAS* and *TP53* or *STK11*, affect TMEs by multiple mutations.

Relationships between pathological subtypes and prognosis were evaluated in a previous study where lepidic was regarded as low-grade, acinar, and papillary as intermediate-grade, and micropapillary and solid as high-grade [49]. It has been reported that the number of FOXP3-positive TILs was higher in invasive pathologic subtypes [34,50], and a high density of stromal FOXP3-positive TILs was associated with shorter recurrence-free probability in stage I adenocarcinomas [50]. The results of our study also showed that significantly more FOXP3-high TILs were observed in invasive histological subtypes than in non-invasive histological subtypes. The concordance rate of FOXP3-positive TILs was 43.7%, and this was not changed by non-intrinsic factors regardless of synchronous or metachronous tumors. There was also no correlation between genetic mutations and FOXP3-positive TILs. It was further clarified that FOXP3-positive regulatory T-cells were strongly associated with pathological subtypes. In contrast to a previous study, the present study found no differences in CD3-positive TILs and the CD8/CD3 ratio between pathologic subtypes [34]. No definitive conclusion has been reached due to few studies regarding the relationship between pathologic subtypes and TMEs being conducted.

This study has several limitations. First, our sample size was relatively small, and the retrospective nature of the study might have induced a selection bias. MPLC is a relatively rare disease, so it is next to impossible to secure enough cases only at a single facility. Therefore, multi-institutional joint study would help to increase the sample size within a certain time frame for a comprehensive investigation. By doing so, more samples with better DNA can be sequenced, resulting in a better understanding of the relationship between TME and mutation profiling. Second, there are no established evaluation methods and cut-off values of CD3-, CD8-, and FOXP3-positive TILs as distinct from PD-L1 assay. Differences in staining intensity due to the antibodies used for the study, selection of the evaluation area, and the pathologists involved would affect the results, particularly scoring of TILs. Therefore, further studies are needed to determine the cut-off value. Third, most C>T/G>A transitions with low variant allelic frequencies were excluded based on strict filtering criteria due to an artifact related to formalin fixation and a lack of normal tissue references. Additionally, given the differences in NGS gene panel content, variant filtering methods, and definitions of TMB thresholds, it is difficult to compare TMB results across studies. However, to our knowledge, this study is the first to examine how various factors impact the TME using MPLC samples.

## Conclusions

Comparing the TME among MPLCs clarified that heterogeneous change of PD-L1 expression was more likely to occur in moderate or heavy smokers and males and confirmed that mutation profiling by deep sequencing plays an important role in understanding the TME. The heterogeneity of regulatory T cells by differences in histological subtypes may impact the effect of immune therapy. Our findings provide deeper insights into the understanding of the heterogeneity of TMEs and for identifying tumors with pre-existing immune activity and biomarkers correlated with responses to ICIs.

## Ethical approval

The study was approved by the institutional review board at Osaka City University Hospital and Wakayama Medical University Hospital, and written informed consent was obtained from all of the patients.

## CRediT authorship contribution statement

**Motohiro Izumi:** Conceptualization, Methodology, Formal analysis, Investigation, Data curation, Writing – original draft, Funding acquisition. **Kenji Sawa:** Investigation, Data curation, Writing – review & editing. **Jun Oyanagi:** Methodology, Investigation, Writing – review & editing. **Ikue Noura:** Investigation. **Mitsuru Fukui:** Formal analysis. **Koichi Ogawa:** Writing – review & editing. **Yoshiya Matsumoto:** Investigation, Writing – review & editing. **Yoko Tani:** Writing – review & editing. **Tomohiro Suzumura:** Writing – review & editing. **Tetsuya Watanabe:** Writing – review & editing. **Hiroyasu Kaneda:** Writing – review & editing. **Shigeki Mitsuoka:** Writing – review & editing. **Kazuhisa Asai:** Writing – review & editing. **Noritoshi Nishiyama:** Writing – review & editing. **Masahiko Ohsawa:** Investigation. **Nobuyuki Yamamoto:** Writing – review & editing. **Yasuhiro Koh:** Conceptualization, Methodology, Supervision, Project administration, Writing – review & editing, Funding acquisition. **Tomoya Kawaguchi:** Conceptualization, Methodology, Project administration, Writing – review & editing, Funding acquisition.

## Declaration of Competing Interest

Y. Koh has received honoraria from Thermo Fisher Scientific. All other authors declare that they have no competing interests.

## Appendix Supplementary materials

Image, application 1

Image, application 2
