# Supplementary material for: Tumor microenvironment disparity in multiple primary lung cancers: Impact of non-intrinsic factors, histological subtypes, and genetic aberrations
Source: Transl Oncol. 2021 Apr 27;14(7):101102. doi: 10.1016/j.tranon.2021.101102 (PMC8102176; doi:10.1016/j.tranon.2021.101102)
Supplement: Supplementary file 1 [file mmc1.docx]

Figure S1


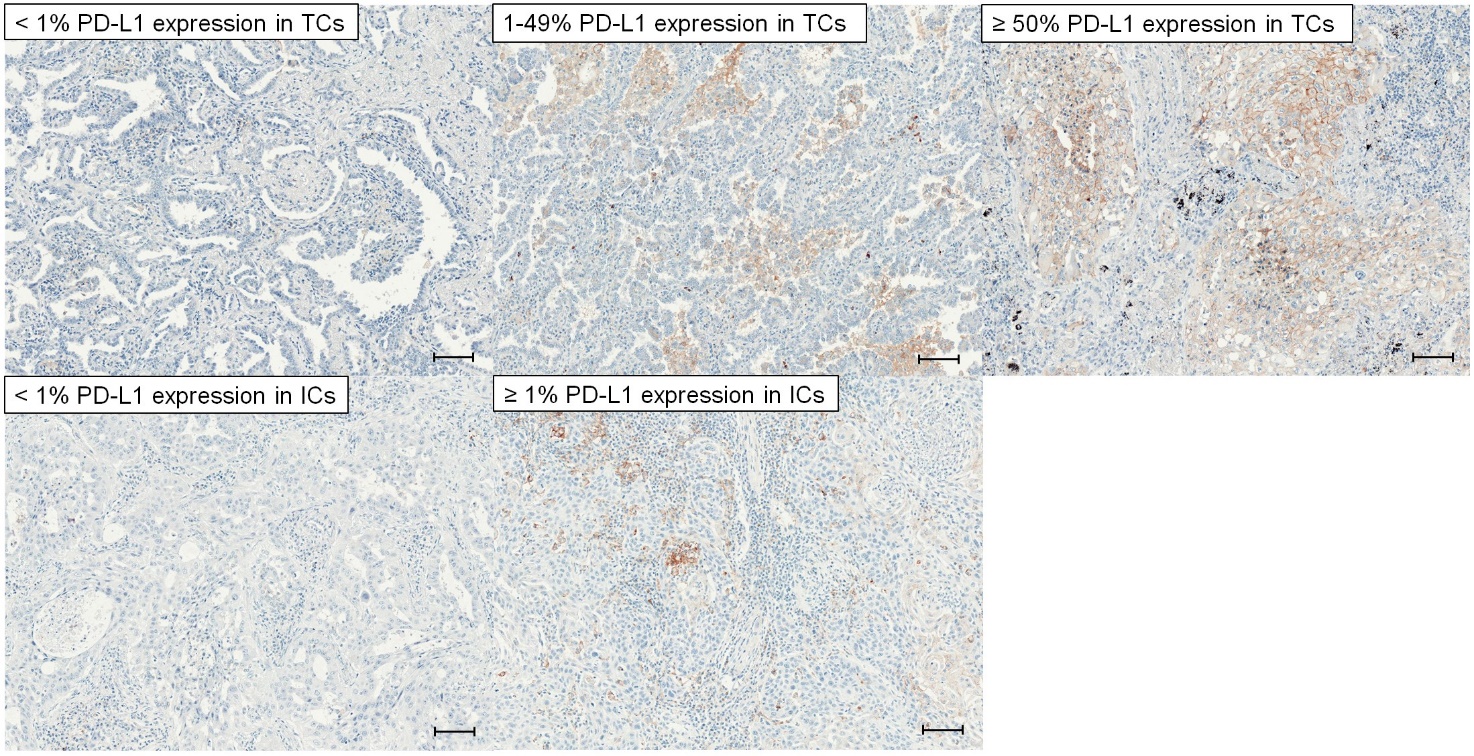


Fig. S1. PD-L1 immunohistochemical analysis. Representative images of < 1% PD-L1 expression in tumor cells (TCs), 1-49% PD-L1 expression in TCs, ≥ 50% PD-L1 expression in TCs, < 1% PD-L1 expression in immune cells (ICs) and ≥ 1% PD-L1 expression in ICs in formalin-fixed paraffin-embedded samples. Scale bar represents 100 μm.

Figure S2


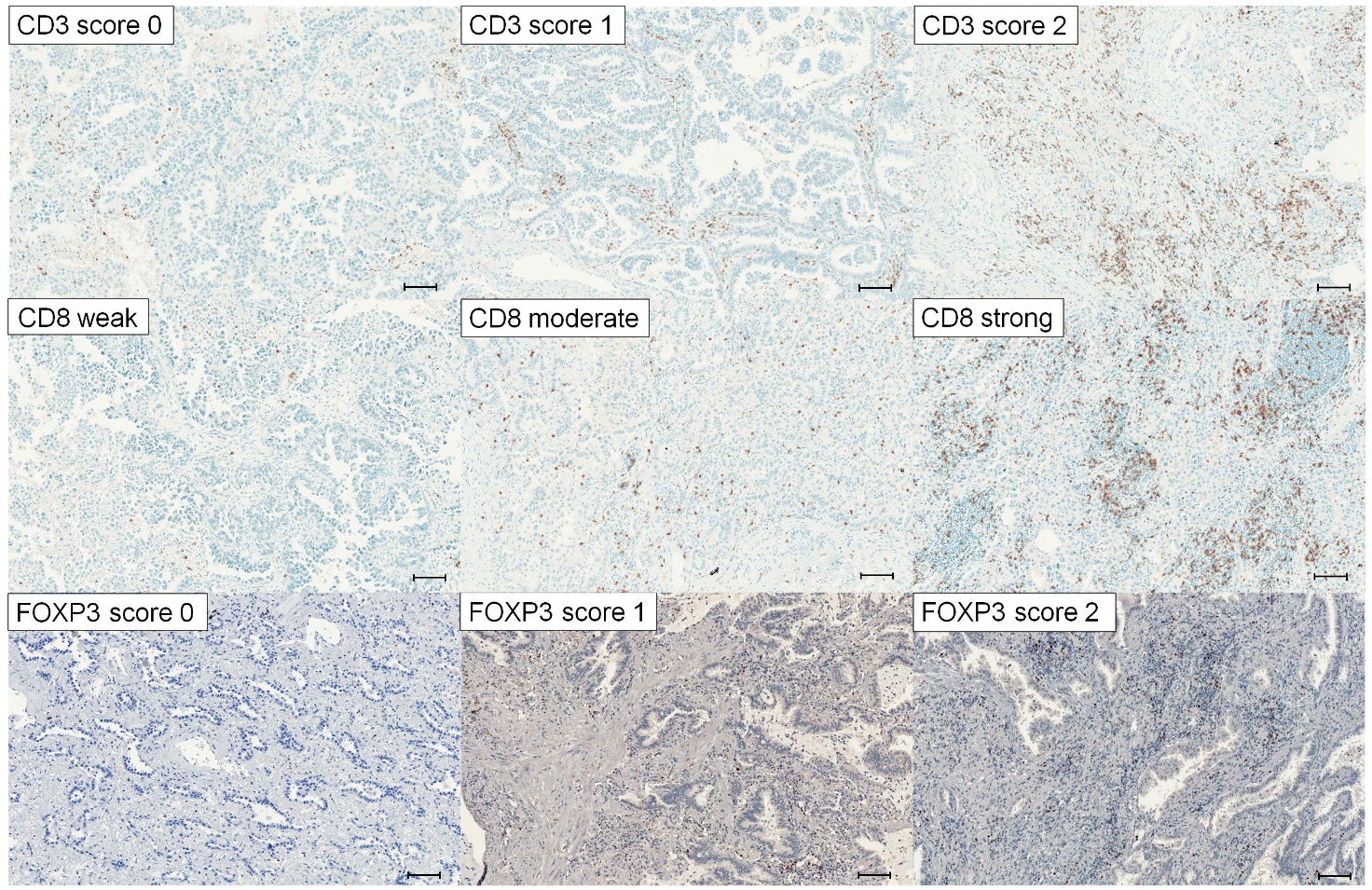


Fig. S2. Immunohistochemical analysis of CD3-, CD8-, and FOXP3-positive tumor infiltrating lymphocytes. Representative images of scoring: 0 (weak), 1 (moderate), and 2 (strong). Scale bar represents 100 μm.

Figure S3


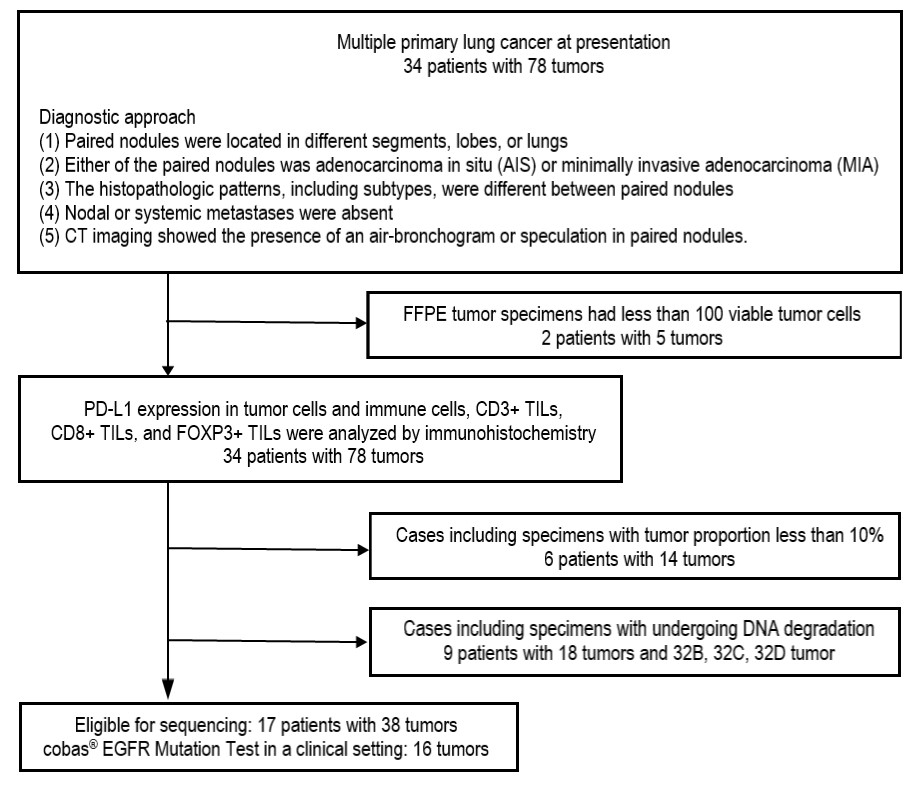


Fig. S3. A flow diagram for patient inclusion in analysis. FFPE, formalin-fixed paraffin-embedded; TILs, tumor infiltrating lymphocytes.

Figure S4


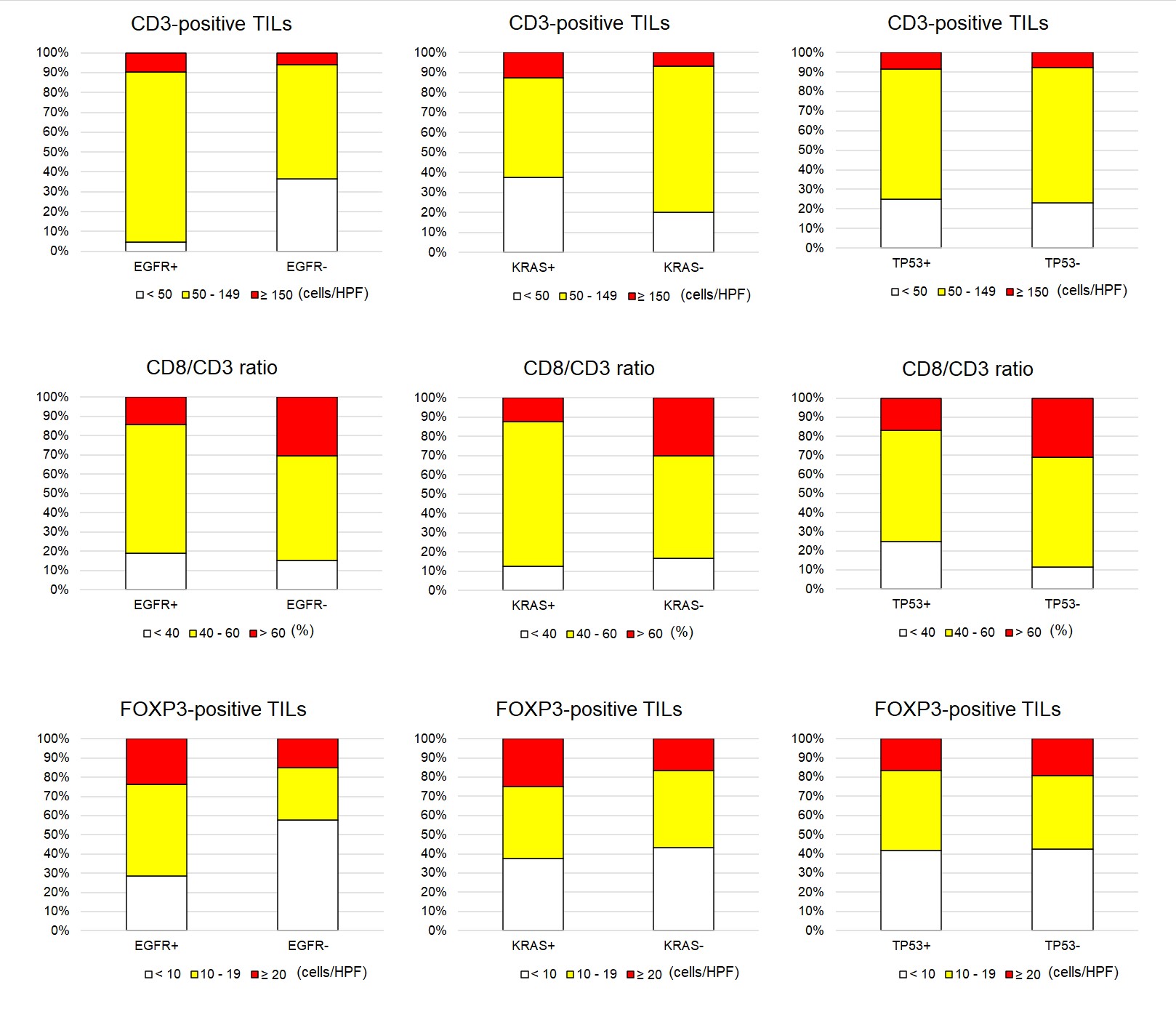


Fig. S4. Correlation between tumor infiltrating lymphocytes (TILs) and genetic aberrations. *EGFR*, *KRAS*, or *TP53* mutations were not involved in TILs by each single mutation.

Figure S5


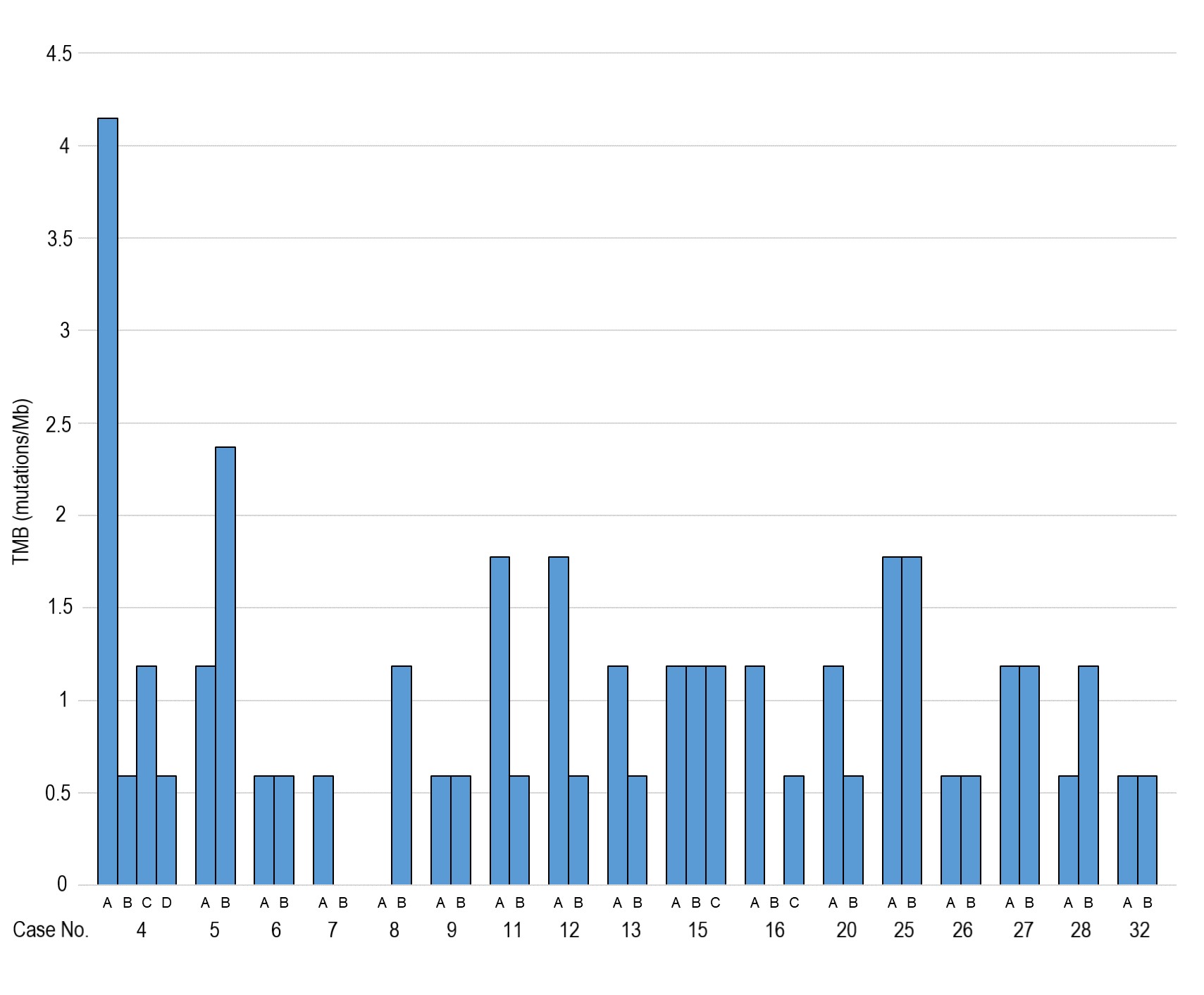


Fig. S5. Tumor mutation burden in each lesion. TMB, tumor mutation burden (mutations/mega-base). The TMB was calculated in 17 cases eligible for next generation sequencing using panel targeting on the 409 cancer-associated genes. TMB ranged from 0 to 4.1 mutations/Mb. TMB had no significant impact on tumor microenvironments.
